# A novel injury paradigm in the central nervous system of adult *Drosophila*: molecular, cellular and functional aspects

**DOI:** 10.1242/dmm.044669

**Published:** 2021-06-01

**Authors:** María Losada-Pérez, Nuria García-Guillén, Sergio Casas-Tintó

**Affiliations:** Instituto Cajal-CSIC, Department of Molecular, Cellular and Developmental Neurobiology, 28002 Madrid, Spain

**Keywords:** JNK, Macrophages, CNS damage, Glia, Immune response, Regeneration

## Abstract

The mammalian central nervous system (CNS) exhibits limited regenerative capacity and the mechanisms that mediate its regeneration are not fully understood. Here, we present a novel experimental design to damage the CNS by using a contusion injury paradigm. The design of this protocol allows the study of long-term and short-term cellular responses, including those of the CNS and the immune system, and of any implications regarding functional recovery. We demonstrate for the first time that adult *Drosophila* *melanogaster* glial cells undergo spontaneous functional recovery following crush injury. This crush injury leads to an intermediate level of functional recovery after damage, which is ideal to screen for genes that facilitate or prevent the regeneration process. Here, we validate this model and analyse the immune responses of glial cells as a central regulator of functional regeneration. Additionally, we demonstrate that glial cells and macrophages contribute to functional regeneration through mechanisms involving the Jun N-terminal kinase (JNK) pathway and the *Drosophila* protein Draper (Drpr), characteristic of other neural injury paradigms. We show that macrophages are recruited to the injury site and are required for functional recovery. Further, we show that the proteins Grindelwald and Drpr in *Drosophila* glial cells mediate activation of JNK, and that expression of *drpr* is dependent on JNK activation. Finally, we link neuron-glial communication and the requirement of neuronal vesicular transport to regulation of the JNK pathway and functional recovery.

This article has an associated First Person interview with the first author of the paper.

## Abstract (precis)

**Summary:** Central nervous system crush injury paradigm in adult *Drosophila* *melanogaster* is a suitable model to study the cellular events, and genetic pathways behind injury responses and functional regeneration. We describe the immune responses of glial cells, neurons and macrophages following injury, and the functional relevance of each response.

## INTRODUCTION

In contrast to the peripheral nervous system (PNS), the mammalian central nervous system (CNS) has limited regenerative capacity (Curcio and Bradke, 2018). Consequently, brain stroke, spinal cord injury or neurodegenerative diseases result in permanent functional impairment. The limited regenerative capacity of neurons, myelin-associated inhibitory factors and the presence of glial scars, restrict regeneration of the mammalian CNS (Cregg et al., 2014; Silver and Miller, 2004). Other vertebrates, such as zebrafish, undergo spontaneous regeneration of the PNS and CNS following injury, thus facilitating functional recovery (Rasmussen and Sagasti, 2017). However, this regenerative capacity deteriorates with age; therefore, as animals age, functional recovery following spinal cord injury is limited (Becker et al., 1997). Invertebrate models, such as *Caenorhabditis elegans* and *Drosophila melanogaster* allow extensive genetic manipulations that contribute to deduce the mechanisms that mediate regeneration of the CNS. Other injury models, such as axon regeneration following laser ablation, have emerged as being suitable to investigate the cellular properties of regeneration (Stone et al., 2010; Yanik et al., 2004). This model has contributed to uncover the implication of microtubules (Chen et al., 2011), dendrite-axon interconversion (Stone et al., 2010) and the decline of spontaneous functional recovery with age (Byrne et al., 2014). However, although these mechanisms are conserved in mammals, they do not participate in CNS regeneration (Curcio and Bradke, 2018).

Neural regeneration studies are primarily focused on axon regeneration as a strategy to restore neural function. The stimulation of damaged neurons has been proposed to overcome deleterious signals originated in injured neurons and neighbouring cells (McQuarrie and Grafstein, 1973; Silver, 2009). Several mechanisms control axonal regeneration, including local cytoskeletal modifications that promote growth cone formation and axon extension (Bradke et al., 2012), axon-soma retrograde signalling (Rishal and Fainzilber, 2014), mitochondrial trafficking (Sheng, 2017), activation of specific regenerative transcriptional programs (Mahar and Cavalli, 2018) and epigenetic modification of selected genes (Weng et al., 2017).

The microenvironment also modulates regeneration following CNS injury. Damaged axons of adult rat CNS neurons regrow when placed in a permissive environment for regeneration (Curcio and Bradke, 2018), an axon laser ablation model in *Drosophila* demonstrated that the glial metabolic status can promote or inhibit axon regrowth (Li et al., 2020). These results suggest a different role of permissive and non-permissive environments for regeneration. Glial cells maintain those neural environments (Sawa et al., 2004). Glial cells proliferate, undergo morphological changes, enwrap axons and engulf cellular debris in response to injury. This process is known as the glial regenerative response (GRR) and it is conserved across species (Kato et al., 2018). GRR can be divided into two, the proliferative response and the immune response (Losada-Perez, 2018). Previous studies in *Drosophila* and mice described a genetic network that regulates the GRR to injury (Kato et al., 2011, 2015; Losada-Perez et al., 2016, 2017). This network includes the genes *kon-tiki/Ng2*, *Notch/Notch1*, *pros/Prox1* and *dorsal/NFkB*, and controls glial cell division and differentiation. GRR has been described in brains of *Drosophila* larvae, although neural trauma in adults is the most common form of CNS injury in humans and, thus, its role during adult CNS damage needs to be investigated.

The GRR immune response involves gene expression of *draper* (*drpr*) and signalling via Jun N-terminal kinase (MAPK8, also known as and hereafter referred to as JNK). *drpr* is the *Drosophila* orthologue of mammalian *Megf10* (*CED-1* in *C. elegans*) (Chiu et al., 2018). Upon neural injury, engulfing cells, macrophages or neighbouring cells remove cellular debris. These cells respond to local cues known as ‘eat-me’ signals released from injured tissue via the Drpr/Megf10/CED-1 receptor (Awasaki et al., 2006; Chiu et al., 2018; Chung et al., 2000; Franc et al., 1996, 1999; Hamon et al., 2006; Kurant et al., 2008; MacDonald et al., 2006).

The engulfment of dying cells in the germ line or epithelial cells is mediated by the phagocytic receptor Drpr through activation of the JNK pathway (Casas-Tintó et al., 2015; Etchegaray et al., 2012). Likewise, mutations of JNK reduce axon regeneration and provoke accumulation of axon debris after injury (Chiu et al., 2018). In addition, knockdown of JNK-pathway components block CED-1 mediated axon regeneration and axon debris removal (Chiu et al., 2018), suggesting a role for JNK pathway in Drpr/CED-1-mediated axon regrowth. This immune system response operates in both *Drosophila* CNS and PNS (Gadani et al., 2015). *Drosophila* adult glial cells become reactive in response to axotomy through JNK-mediated upregulation of *drpr* (MacDonald et al., 2013). In addition to the glial immune response, the immune system also contributes to regeneration of the CNS following injury. Studies in vertebrates revealed the role of macrophages during wound healing, debris removal or through the recruitment of other cells to the injury site (Brancato and Albina, 2011; Koh and DiPietro, 2011; Werner and Grose, 2003). However, little is known about the role of macrophages after neural injury in *Drosophila*.

Some vertebrates recover locomotion following limb amputation by adapting the activity of the central pattern generator (CPG), a feature that illustrates the robustness of the locomotor system (Isakov et al., 2016; Kirpensteijn et al., 1999; Mendes et al., 2014). To identify bona fide mechanisms of neural regeneration it is, therefore, necessary to find a reliable method to investigate injured-limb movement instead of the locomotor capacity of the complete animal. Here, we present a novel paradigm of injury in adult flies, which reproduces the main features described in other animal models after CNS injury and allows the study of functional regeneration in a temporally restricted manner. We describe for the first-time the spontaneous functional regeneration following injury in adult *Drosophila*, the activation of immune components, such as Drpr or JNK in neural cells and the requirement of macrophages for functional recovery.

Therefore, application of crush injury is a suitable technique to study cellular events and genetic pathways to understand responses of the CNS to injury and their relevance for functional recovery.

## RESULTS

### A procedure for adult crush injury

**Fig. 1.**
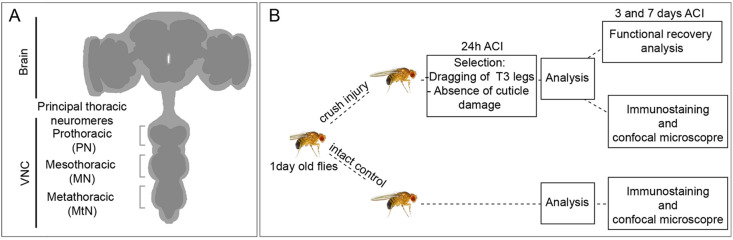
**Crush injury protocol.** (A) Schematic representation of adult *Drosophila* CNS showing the main thoracic neuromeres. (B) Diagram of standard protocol followed in this work.

To study GRR in adult flies we used forceps to apply pressure from the exterior to damage the metathoracic segment of adult flies (1 day after eclosion), leading to impaired movement of T3 legs (innervated by the metathoracic ganglia) and locomotor dysfunction. To cause the injury, we first anesthetised the animal on a CO_2_ plate, ventral side up, and gently pinched the thorax with forceps pointing to the metathoracic segment (Fig. 1A). As the injury was performed manually, variability of the damage was expected to be broad. To reduce variability, we classified injured flies 24 h after crush injury (ACI) (unless otherwise indicated) and selected only individuals that were dragging the third pair of legs. In contrast to other injury methods, crush injury allows the study of functional recovery as the cuticle and legs remain intact (Fig. 1B).

**Fig. 2.**
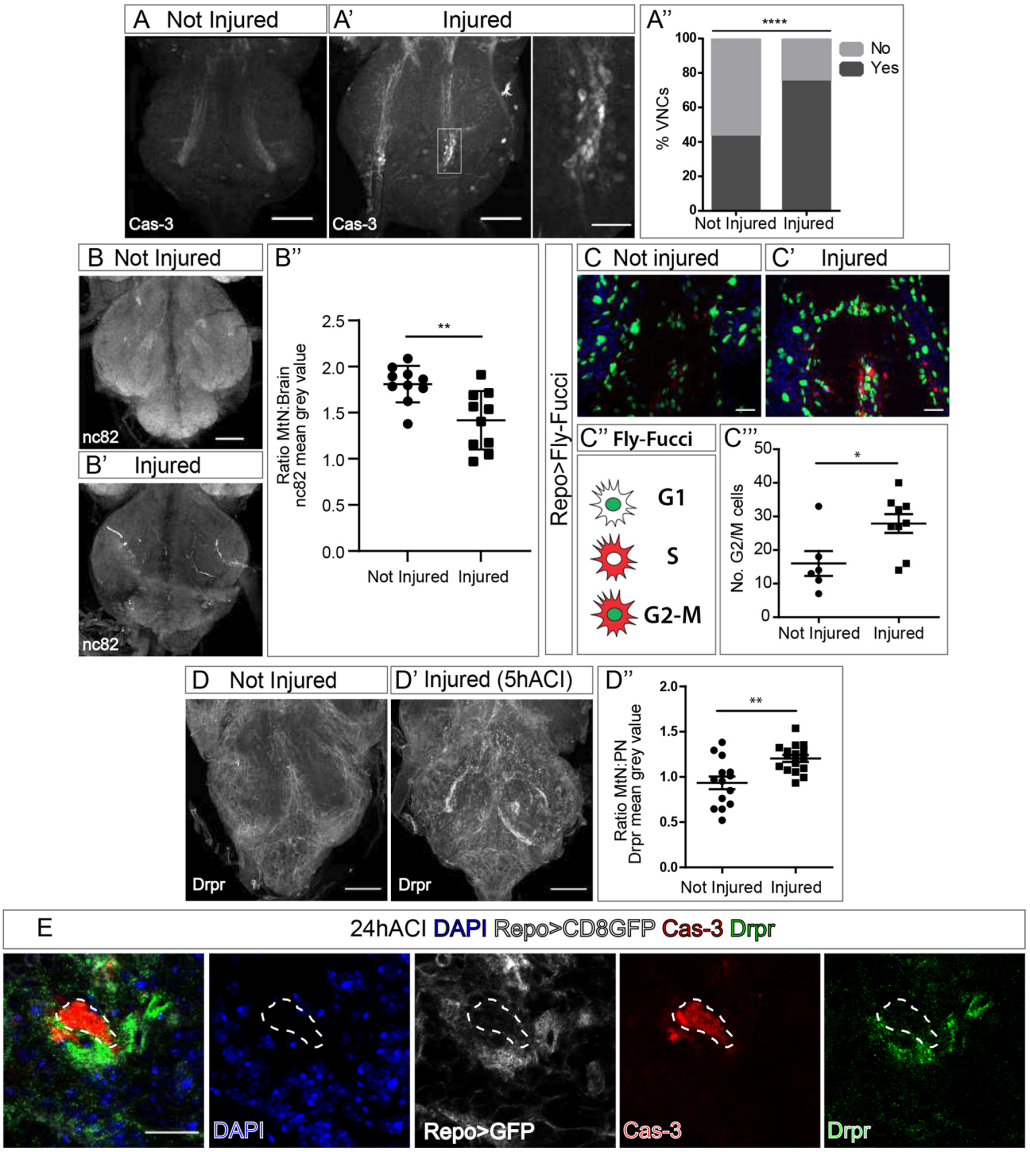
**Crush injury can be used to study GRR.** (A,A′) Representative metathoracic neuromeres (MtNs) showing anti-caspase-3 staining in not injured (A) and injured (24 h ACI) VNCs (A′). The boxed area in A′ is shown magnified in the most-right image. (A′′) Binomial test, comparing the percentage of injured and not injured VNCs (%VNCs) with caspase-3 (Cas-3)-positive signal (Yes), and VNCs without Cas-3-positive signal (No). ****P<0.0001. (B,B′) Representative images of not injured (B) and injured (B′, 24 h ACI) VNCs stained for Bruchpilot (nc82). (B″) Quantification of the MtN:Brain nc82 ratio mean intensity signal. Unpaired *t*-test, **P<0.005. (C,C′) Representative images showing Fly-FUCCI in glial cells of not injured (C) and injured (C′, 24 h ACI) VNCs. (C″) Schematic of Fly-FUCCI corresponding to phases of the cell-cycle. (C‴) Quantification of cells positive for GFP and RFP in G2/M phase. Unpaired *t*-test, *P<0.05. (D) Drpr staining of not injured (D) and injured (D′, 5 h ACI) VNCs. Quantification of the MtN:PN mean grey value ratio of the Drpr signal (D″). Unpaired *t*-test, **P<0.005. (E) Images of cell samples 24 h ACI stained for Drpr (green) colocalising with glial membrane (Repo>myrRFP, grey) and surrounding the cell debris (stained for Cas-3, red) 24 h ACI. Areas surrounded by dashed lines indicate the Cas-3 signal. Nuclei were stained with DAPI (blue). Genotypes: wild type (A,B,D); *repoGal4>UAS-Fly-FUCCI* (C); *repoGal4>UAS-myrRFP* (E). Scale bars: 50 μm (A,B,D); 15 μm (A′ right panel, C,E).

To validate the injury site, we determined cell damage in the CNS through staining for caspase-3 to detect cell death (Sarkissian et al., 2014) (Fig. 2A,A′). In most injured ventral nerve cord**s** (VNCs) we found caspase-3-positive signals, as this feature is statistically less common in non-injured controls (Fig. 2A″). To ensure that the caspase-3 signal correlates with programmed cell death, we examined whether cells with caspase-3-positive signal correspond with condensed nuclei (Lu et al., 2005; Toné et al., 2007). We measured the DAPI signal in caspase-3-positive and caspase-3-negative nuclei of injured VNCs, and found that the average intensity of DAPI signals in caspase-3-positive nuclei is significantly higher compared to caspase-3-negative nuclei (Fig. S1A). Thus, we conclude that the caspase-3 signal we observed in injured VNCs corresponds to cell death.

Next, to demonstrate that this novel crush injury produces cellular damage, we analysed the synapses in the VNC. Bruchpilot (Brp, also known as and hereafter referred to as nc82) is a presynaptic protein located in the active zones (Jordán-Álvarez et al., 2017), and one of the last proteins incorporated to synapses (Fouquet et al., 2009). Therefore, nc82 staining is a reliable marker for mature synapses, indicating the cellular state of the neuropil. nc82 immunostaining of VNCs 24 h ACI revealed a decrease of the nc82 signal in injured animals compared to non-injured controls of the same age (Fig. 2B,B′). We observed that the nc82 signal in the non-injured area of the VNC also decreased, probably due to synapse loss of dying neurons within the injured area that project to other areas. Therefore, to normalise the nc82 signal, we calculated the ratio of metathoracic neuromeres to brain (MtN:Brain) in each fly, corresponding to the ratio of injured to intact area. These results suggest that the injury provokes a reduction of the number of synapses (Fig. 2B″), consistent with the lack of movement of the third pair of legs observed after crush injury (Movie 1).

To determine the extent of the damage caused by crush injury, we analysed the cuticle and muscles in the area of damage. To avoid selecting flies with unwanted damage, e.g. bacterial infection, we discarded those flies that 24 h ACI showed any sign of undesirable external damage, such as deformation or change of pigmentation (Fig. S1B). To determine whether our injury paradigm results in muscle damage, we dissected the thorax of adult flies 24 h ACI. We damaged flies expressing green fluorescent protein (GFP) in muscle cells (*mhc*-Gal4) to visualise the ultrastructure of their metathoracic dorsoventral muscle, which are in the damaged area. Confocal images show that crush injury causes noticeable fibre disruption and damage to this muscle (Fig. S1C). We also investigated whether the integrity of injured VNCs remained intact. Although we found disruption of the blood brain barrier (Fig. S1D), we did not observe defects in the macrostructure of the VNC (Fig. S1E,E′). Therefore, we concluded that there is substantive damage in both the CNS and metathoracic dorsoventral muscles upon crush injury, comparable to what happens to humans with spinal cord injuries (Biering-Sørensen et al., 2009; Emery et al., 1998; Kakulas, 1999; Norenberg et al., 2004).

To elucidate whether this new paradigm produces the stereotypical responses of glial cells described in previous reports (Griffiths and Hidalgo, 2004; Kato et al., 2009, 2011; Losada-Perez et al., 2016; Purice et al., 2017; Smith et al., 1987; Treherne et al., 1984), we first determined whether crush injury induced proliferation of glial cells. To visualise cell-cycle progression and to quantify cell proliferation, we applied a 24-h pulse with the thymidine analog bromodeoxyuridine (BrdU). Quantification of BrdU-positive cells in the metathoracic region revealed significantly increased BrdU incorporation in glial cells (Repo^+^) upon injury (Fig. S1F,F″). To further confirm glial cell proliferation in response to crush injury, we visualised the fluorescent ubiquitination-based cell-cycle indicator (FUCCI) cell-cycle reporter (Zielke et al., 2014). This Fly-FUCCI reports accumulation of E2F (GFP) and CycB (RFP) protein, indicating phases G1, S or G2/M of the cell cycle. We restricted expression of this UAS-reporter to glial cells under the control of the *repoGal4* promoter (Casas-Tintó et al., 2017) and quantified the number of RFP- and GFP-double-positive (RFP+GFP+) cells to determine mitotic events. Our analysis showed that crush injury induces glial cell proliferation (Fig. 2C,C″).

It is well-documented that expression of the engulfment factor *drpr* as well as engulfment of cellular debris are features of GRR (Doherty et al., 2009, 2014; Logan et al., 2012; MacDonald et al., 2006, 2013). To determine the amount of Drpr in glial cells after injury, we used antibody specifically recognising Drpr to immunostain dissected VNCs 5 h ACI. Compared to that of controls, the Drpr signal is significantly higher in the metathoracic ganglia of injured VNCs (Fig. 2D,D′, Fig. S2A). We calculated the ratio between MtN and prothoracic neuromere (MtN:PN), which corresponds to the ratio of injured to non-injured area. The results indicate that, upon crush injury, Drpr protein levels were higher in the injured area (Fig. 2D″). Subsequently, 24 h ACI the Drpr signal colocalised with glial membranes that surround caspase-3-positive bodies (Fig. 2E, Movie 2). These results indicate that crush injury can activate the phagocytic response in glial cells of the CNS.

Next, we evaluated functional recovery following crush injury. Previous work in *Drosophila* larvae demonstrated that GRR is a natural mechanism that promotes tissue repair (Kato et al., 2011). However, to date, there is no paradigm that uncovers functional recovery in adult flies. We, therefore, performed a functional assay to investigate whether this tissue repair can be translated into a functional recovery in *Drosophila* adults. We monitored locomotion of injured flies and analysed the movement of the damaged legs; i.e. we selected injured flies that dragged rear T3 legs 24 h ACI (T0) (Movie 1) and, subsequently, analysed the movement at 3 and 7 days ACI in comparison with T0.

**Fig. 3.**
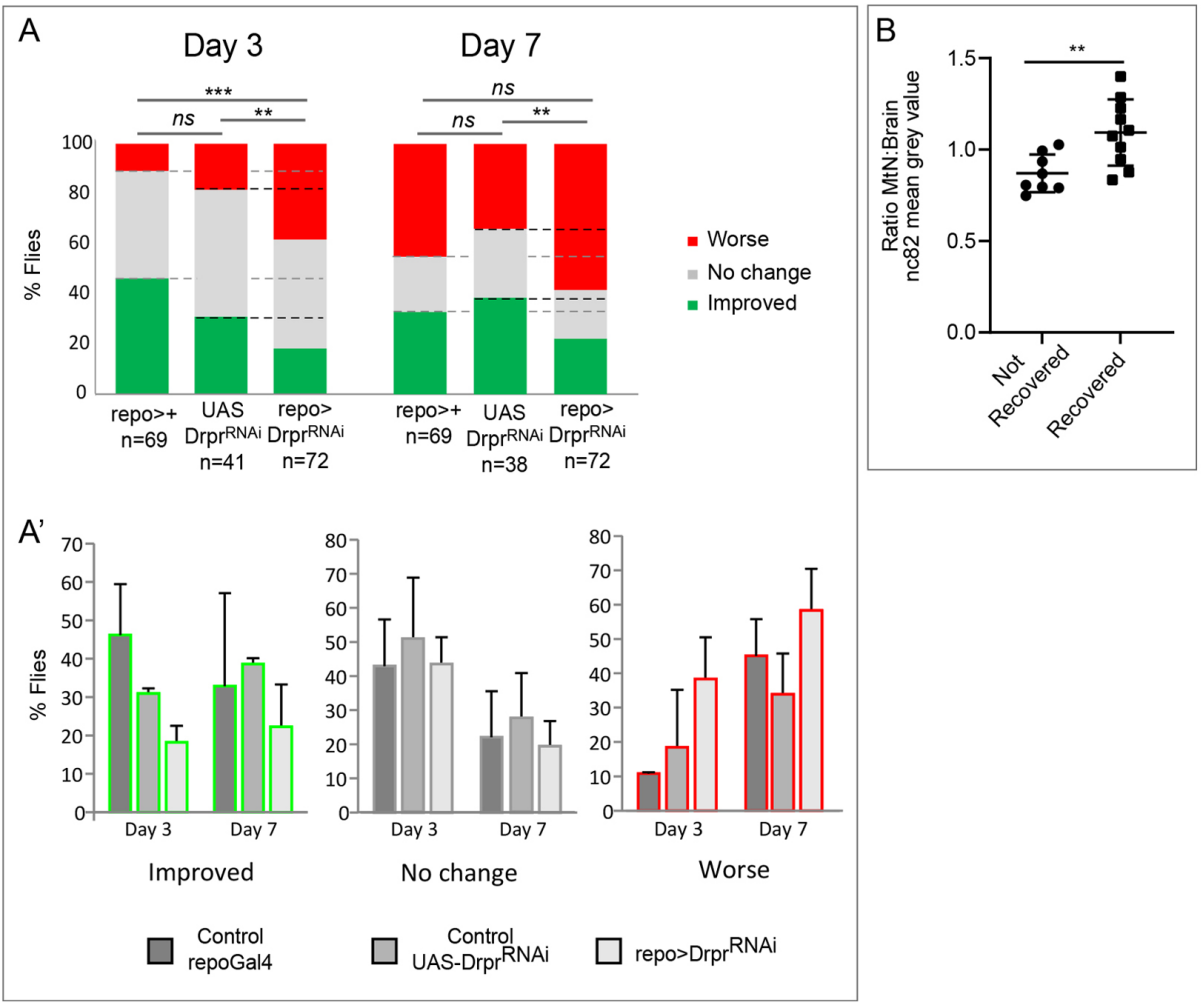
**Adult *Drosophila* recover functionality after CNS crush injury and this requires glial Drpr.** (A) Histograms representing the percentage of injured flies that correspond to each of the three movement categories Worse (red), No change (grey) or Improved (green), compared to T0 (24 h ACI). Functional recovery capacity of control flies (*repoGal4* and *UAS-drprRNAi* alone) compared with *drpr* knockdown in glia. Chi-square test with two degrees of freedom, ***P<0.0001, **P<0.005. (A′) Histograms represent the percentage of flies corresponding to each category separately. (B) Quantification of the MtN:Brain nc82 mean intensity signal ratio of not recovered and recovered flies 7 days ACI. Unpaired *t*-test, **P<0.005. Genotypes: *tubGal80ts*, *repoGal4/+*, UAS *DrprRNAi/+*, *tubGal80ts*, *repoGal4>UAS-DrprRNAi* (A,A′); wild type (B).

These results demonstrate that injured flies recover the movement of the legs at 3 and 7 days ACI (Fig. 3A,A′). To demonstrate that functional recovery implies CNS regeneration, we quantified the number of synapses within the injured area by staining for nc82 at 7 days ACI. We compared the ratio of the MtN:Brain nc82 signal in recovered and not recovered flies, and found it to be higher in recovered flies (Fig. 3B). These results demonstrate for the first time that functional recovery following CNS injury in *Drosophila* correlates with synapse number in the CNS. Additionally, we observed that the MtN:Brain ratio of the nc82 signal in recovered and non-recovered flies is lower than at 24 h ACI (Fig. 2B″), which suggests that the loss of synapses is most likely to occur 7 days ACI.

Next, we aimed to study the mechanisms that govern neural repair. Previous work has focused on the proliferative component of GRR; however, we hypothesise that immune components play a central role in neural repair. We focused our initial efforts on Drpr, as this protein is one of the most-extensively studied components of the immune system in injury models (Doherty et al., 2014; Logan et al., 2012; MacDonald et al., 2013; Purice et al., 2017). We observed that *drpr* is consistently upregulated in glial cells within the injured area (Fig. 2D″) – in line with previous reports regarding other injury models (Doherty et al., 2009, 2014; Logan et al., 2012; MacDonald et al., 2006, 2013). Next, we tested whether expression of *drpr* in glial cells is required for functional recovery following crush injury. By using RNA interference (RNAi), we downregulated expression of *drpr* in glial cells (*repo-*Gal4) and analysed leg movement. The results show that downregulation *drpr* in glial cells (repo>*drpr^RNAi^*) prevented the spontaneous functional recovery of the movement (Fig. 3A,A′). To further validate the contribution of Drpr, we tested the functional recovery in *drpr* mutant flies (*draper Δ5* null allele) and obtained similar results (Fig. S3A,B). These results highlight that manipulation of gene expression in glial cells is sufficient to modify functional recovery, and that Drpr is required for functional recovery following CNS crush injury.

### Drpr and JNK in crush injury

**Fig. 4.**
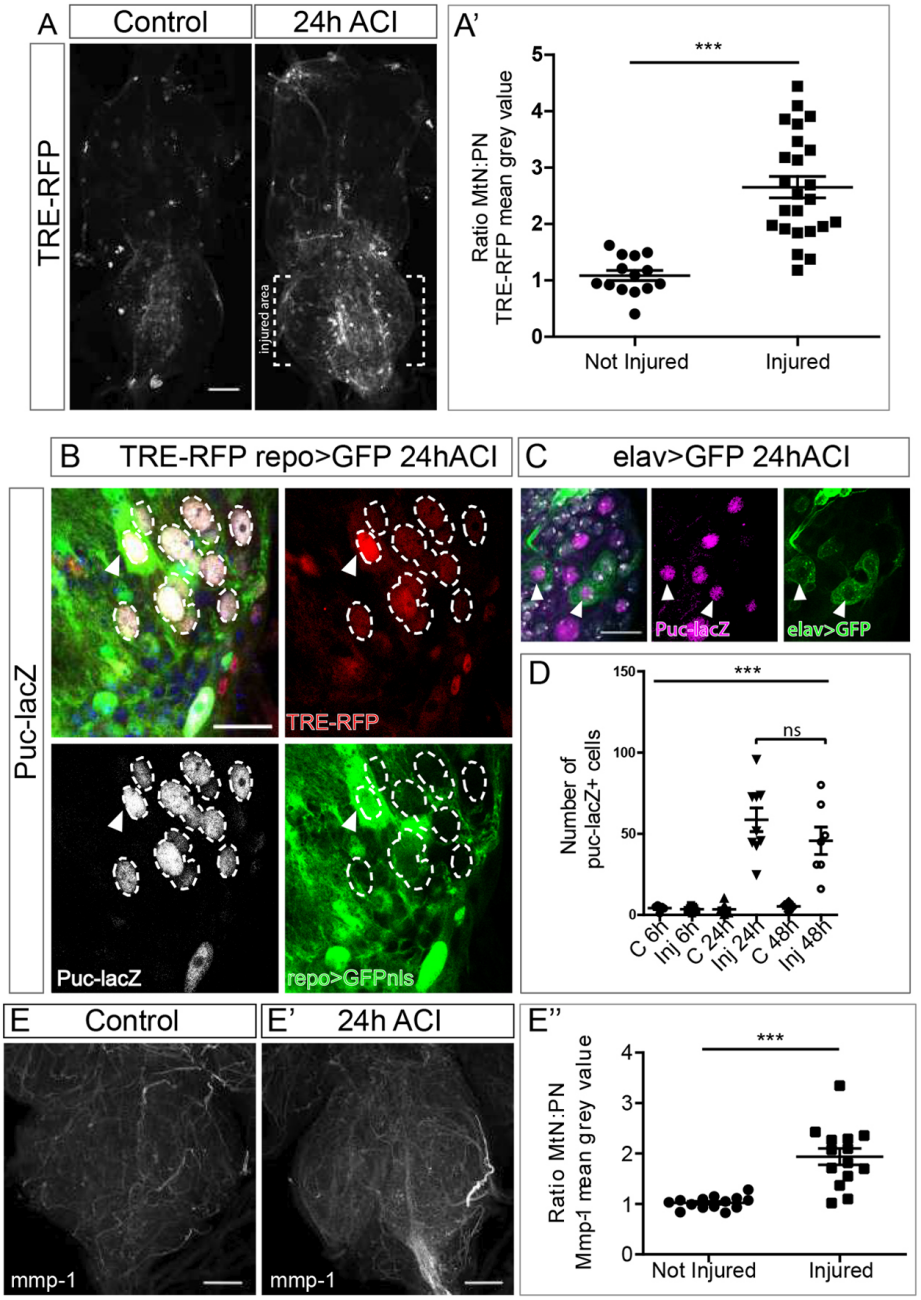
**The JNK signalling pathway is activated in glial cells upon crush injury.** (A) Representative images of not injured and injured VNCs showing the TRE-RFP reporter signal. (A′) Quantification of activated JNK signalling. Plotted is the metathoracic neuromere to prothoracic neuromere (MtN:PN) ratio of the TRE-RFP mean grey value of not injured to injured VNCs. Unpaired *t*-test, ***P<0.0001. (B) Images show a representative section of an injured MtN expressing the JNK reporters TRE-RFP and puc-Z, and the glial marker repo>GFP. puc-Z-positive cells, surrounded by dashed lines, that are also positive for TRE-RFP. Arrowheads indicate glial cells with active JNK. (C) Representative section of an injured MtN marked with the JNK reporter puc-Z and the neuronal marker elav>GFP. Arrowheads indicate neurons with active JNK. (D) Plotted is the number of puc-Z-positive cells in not injured and injured VNCs at different time points. One-way ANOVA, ***P<0.0001; Bonferoni's multiple comparison; n.s. not significant. (E-E′) Representative images of MtNs stained for Mmp-1 in not injured (E) and injured (E′; 24 h ACI) VNCs. (E′′) Quantification of the MtN:PN ratio of the Mmp-1 signal mean grey value. Unpaired *t*-test, ***P<0.0001. Genotypes: *TRE-RFP; repoGal4>UAS-GFPnls* (A,E). *TRE-RFP; repoGal4>UAS-GFPnls/puckered-LacZ* (B). *elavLexA>lexAOP-GFP/+* (C). Scale bars: 50 μm (A,E), 15 μm (B,C).

Drpr signalling has been studied in response to degenerating axons in alternative injury paradigms in *Drosophila* adult CNS; Drpr activates downstream pathways via Stat92E, MMP-1 or JNK (Doherty et al., 2014; MacDonald et al., 2013; Purice et al., 2017). Moreover, axotomy of legs or wings stimulate the activity of the AP-1/JNK pathway in the VNC (Purice et al., 2017). We analysed JNK activation in response to crush injury by using the AP1-recognition sequence TPA responsive element (TRE) conjugated to red fluorescent protein (RFP) (Chatterjee and Bohmann, 2012). This TRE-RFP signal was detected in the entire VNC in both injured as well as non-injured VNCs, with levels significantly increased in the injury area of damaged VNCs (Fig. 4A). To validate this observation, we calculated the MtN:PN ratio of RFP, which confirmed the increase of TRE-RFP reporter signal in injured VNCs (Fig. 4A′). To further confirm JNK activation, we used an additional JNK reporter, *puckered-lacZ* (puc-Z) (Martín-Blanco et al., 1998). To validate the reporter proteins, we first showed that all puc-Z-positive cells are also TRE-RFP positive (Fig. 4B). We also observed that JNK activation following injury occurs in both neurons and glial cells (Fig. 4B,C). Finally, we analysed the temporal activation of the JNK pathway in response to crush injury. We quantified JNK activation 6, 24 and 48 h ACI, and found a significant increase in the number of puc-Z-positive cells 24 h ACI, which is maintained up to 48 h ACI (Fig. 4D).

In addition, we analysed the protein expression of matrix metalloproteinase 1 (MMP1), which is directly associated to JNK activity (Purice et al., 2017). We used a specific anti-MMP1 antibody and stained injured and non-injured VNCs. Quantification of the MMP1 signal showed an increase in the injured area (Fig. 4E-E″, Fig. S2B), in line with previous results. Thus, we conclude that crush injury activates JNK signalling in the CNS.

**Fig. 5.**
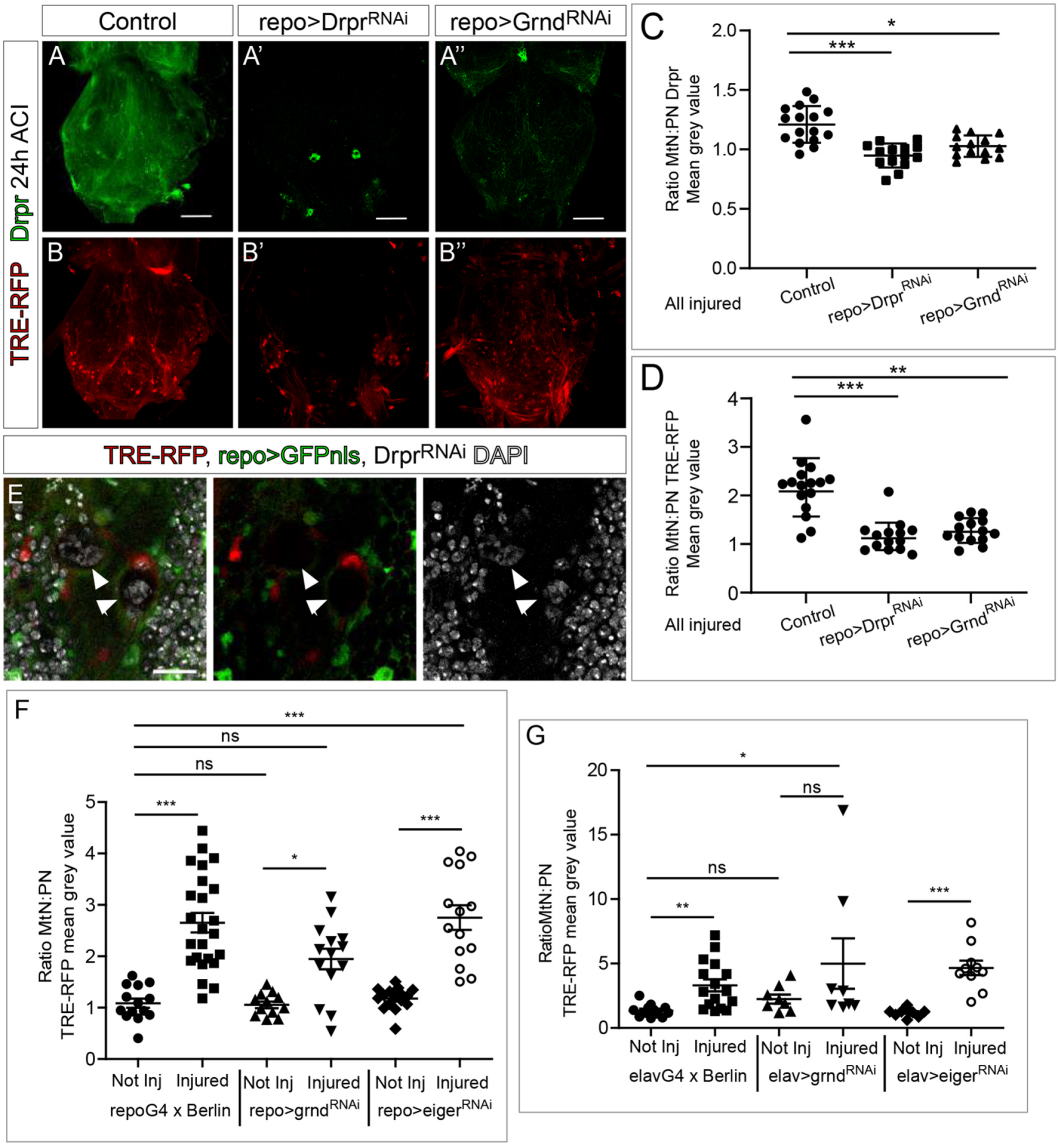
**JNK and** Drpr **regulate each other and this requires Grnd.** (A-B″) Representative metathoracic neuromeres (MtNs) stained for Drpr, showing the TRE-RFP reporter signal at 24 h ACI in control flies (A,B) and in flies with knocked down *drpr* (A′,B′) or knocked down *grnd* (A″,B″) in glial (*repoGal4*) cells. (C) Quantification of MtN:PN ratio of Drpr staining. One-Way ANOVA, *P<0.05; Bonferroni multiple comparison, ***P<0.0001. (D) Quantification of MtN:PN ratio of the TRE-RFP reporter signal. One-Way ANOVA, **P<0.001; Bonferroni multiple comparison, ***P<0.0001. (E) Images show the accumulation of nuclear debris (arrowheads, stained with DAPI, grey) due to downregulation of *drpr* expression in glial cells. The TRE-RFP JNK reporter is shown in red, glial cells (*repo*>*GFP*) are shown in green. (F,G) Quantification of the metathoracic neuromere to prothoracic neuromere (MtN:PN) ratios of the TRE-RFP mean grey value of not injured to injured VNCs after knocking down *grnd* or *eiger* in glial cells (*repoGal4*, F) or neurons (*elavGal4,* G). Dunn's multiple comparison test, *P<0.05, **P<0.001, ***P<0.0001. ns, not significant. Genotypes: *TRE-RFP; repoGal4-UAS-GFPnls* (A-D). *TRE-RFP; repoGal4>UAS-GFPnls, UAS-drprRNAi* (A′,B′,C-E). *TRE-RFP; repoGal4>UAS-GFPnls, UAS-grndRNAi* (A″,B″,C,D). *TRE-RFP; repoGal4>UAS-*GFPnls, *TRE-RFP; repoGal4>UAS-GFPnls, UAS-grndRNAi*, *TRE-RFP; repoGal4>UAS-GFPnls, UAS-eigerRNAi* (F). *TRE-RFP; elavGal4>+, TRE-RFP; elavGal4>UAS-grndRNAi, TRE-RFP* and *elavGal4>UAS-eigerRNAi* (G). Scale bars: 50 μm (A,B), 15 μm (E).

Next we asked whether activation of JNK signalling is triggered by Drpr, as described in other axotomy paradigms (MacDonald et al., 2013; Purice et al., 2017). Expression of *drpr* small interfering RNA (siRNA) reduced the Drpr protein signal in glial cells (we were unable to detect Drpr staining except in two neurosecretory neurons within the MtN) and prevented the increase of Drpr signalling (MtN:PN ratio) upon injury in the VNC (Fig. 5A,A′,C and Fig. S2C). Similarly, we analysed the activity of the JNK pathway – by using the TRE-RFP reporter – in injured animals upon *drpr* knockdown in glial cells. Quantification of the TRE-RFP signal showed a significant reduction of JNK activation upon injury when compared to VNCs of control injured flies (Fig. 5B,B′; Fig. S2D). In line with these results, the increase of TRE-RFP reporter signal ratio (MtN:PN ratio) upon injury was prevented (Fig. 5D). We also observed spots of DNA accumulation in the injured area, suggesting the presence of apoptotic cellular debris due to the absence of Drpr (Fig. 5E). These results indicate that Drpr is required for JNK activation, in line with previous reports in other injury models.

The JNK pathway is evolutionarily conserved from fruit fly to humans (La Marca and Richardson, 2020; Pinal et al., 2019; Wu et al., 2019; Zeke et al., 2016). JNK signalling can be triggered by various extrinsic and intrinsic cues, and regulates a wide range of cellular process including proliferation, differentiation, migration and cell death (Weston and Davis, 2007). In *Drosophila*, binding of the tumor necrosis factor (TNF) orthologue Eiger (Egr) to its receptor Grindelwald (Grnd) activates JNK signalling pathway (Andersen et al., 2015; Glise et al., 1995; Igaki et al., 2002; Moreno et al., 2002; Portela et al., 2019a; Takatsu et al., 2000; Wu et al., 2019). To determine the contribution of the canonical ligand Eiger and the receptor Grnd in JNK pathway activation after crush injury, we downregulated expression of each, specifically in neurons or glia, and measured JNK pathway activation (assessed by measuring the MtN:PN ratio of TRE-RFP reporter). The results showed that *grnd* expression is required for JNK pathway activation in both neurons and glia, although the difference between non-injured and injured VNCs in *repo>grnd^RNAi^* was still significant (Fig. 5F). Thus, to confirm the requirement of Grnd in glial cells, we expressed the dominant-negative form of Grnd, Grnd-Extra (Andersen et al., 2015). JNK activation (MtN:PN ratio) after injury was significantly decreased upon expression of Grnd-Extra in glial cells compared with controls (Fig. S2E). To further confirm the presence of Grnd in the injured area, we stained adult brains with a specific antibody against Grnd. Quantification of confocal images showed that Grnd is located in the adjacent region between the glial and neuron membrane (Fig. S2F,F′). By contrast, *RNAi* of *eiger* in glial cells or neurons did to not affect JNK activation in VNCs upon injury (Fig. 5F,G). To confirm the implication of Eiger in JNK pathway activation in response to crush injury, we analysed different *eiger* mutants that had recently been validated (Kodra et al., 2020). The results showed a similar degree of JNK activation compared to that in controls (Fig. S2G) and suggest that, in this context, Eiger is not required for the activation of JNK pathway. Activation of JNK signalling through the Grnd receptor – but in a way that is independent of its ligand Eiger – has been previously described in response to perturbation of cell polarity in epithelial tumor model (Andersen et al., 2015).

Moreover, the upregulation of *drpr* by JNK has previously been described in *Drosophila* (axotomy paradigms) and in *C. elegans* (Chiu et al., 2018; MacDonald et al., 2013). Therefore, we aimed to determine whether activity of the JNK pathway activates *drpr* expression through activation of the Grnd receptor. To reduce JNK pathway activity, we downregulated *grnd* by using RNAi specifically in glial cells (Fig. 5A,B″). We stained injured VNCs with anti-Drpr (Fig. 5A,A″), quantified the Drpr signal (Fig. S2C) and calculated the Drpr MtN:PN ratio (Fig. 5C). The results showed a reduction of Drpr signal in injured animals when *grnd* gene expression was downregulated, suggesting that JNK pathway activity in glial cells is required for upregulation of *drpr* expression upon injury in the VNC (Fig. 5A,A″,C). To validate these results, we reproduced similar experiments after overexpression of Grnd-Extra (Andersen et al., 2015) in glial cells, and obtained similar results (Fig. S2H). Together, our results indicated that *drpr* is upregulated in glial cells of the VNC upon crush injury, leading to activation of JNK signalling in glial cells and neurons via the Grnd receptor. We, therefore, concluded that JNK pathway activation in glial cells promotes *drpr* expression and vice versa.

### Neuronal vesicular transport is required for regulation of JNK signalling and functional recovery upon injury

**Fig. 6.**
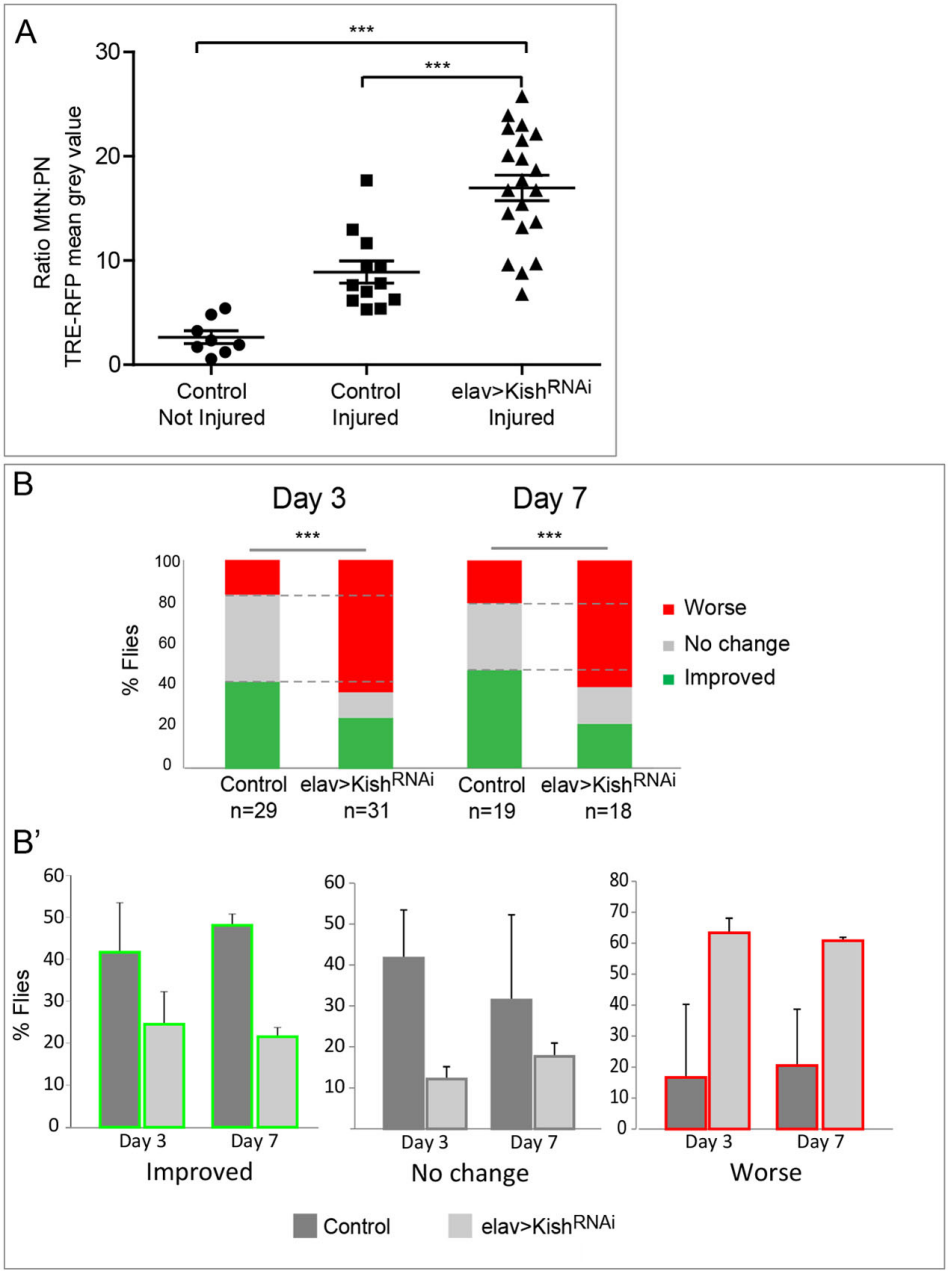
**Vesicular trafficking in neurons** **is required for JNK regulation and functional recovery.** (A) Plotted is the metathoracic neuromere to prothoracic neuromere (MtN:PN) ratio of the TRE-RFP mean grey value of not injured and injured control flies, and injured flies whose vesicular transport in neurons is blocked. Dunn's multiple comparison, ***P<0.0001. (B) Percentage of functional recovery capacity of *kish* knockdown flies compared to that of controls. Chi-square test with two degrees of freedom, ***P<0.00001. (B′) Histograms represent percentage of flies in each category of movement, i.e. Worse (red), No change (grey) or Improved (green). Genotypes: *TRE-RFP*, *tubGal80ts; UAS-KishRNAi/+* (Control) and *TRE-RFP*, *tubGal80ts;* elavGal4>*UAS-KishRNAi* (A,B).

As glial cells receive signals from injured tissue, we hypothesised that affected neurons upon injury increase vesicular trafficking and secretory activity. To investigate whether the secretory nature of neurons implicated neuron-glial communication in this injury paradigm, we knocked down *kish* expression in neurons, which is reported to be involved in vesicle transport and secretion (Gershlick et al., 2016; Portela et al., 2019b; Wendler et al., 2010). We analysed JNK signalling following injury in these animals (*elav>kish^RNAi^*) and found that knockdown of *kish* stimulates JNK pathway signalling (Fig. 6A). However, *kish* knockdown in glial cells (*repo>kish^RNAi^*) did not affect activation of the JNK pathway in injured VNC (Fig. S4). These results suggested that secretion from neurons and, therefore, neuron-glial communication is required for JNK regulation in response to CNS injury.

To validate the relevance of neuron-glial communication in the activation of glial responses following injury, we next analysed functional recovery of injured flies after blocking vesicular transport in neurons. The results showed that functional recovery was compromised upon *kish* knockdown in neurons (Fig. 6B,B′). Hence, we concluded that, in neurons, the reduction of vesicular transport in response to *kish RNAi* is detrimental for functional recovery upon crush injury.

Taken together, these results highlight that neuron-glial communication involving vesicular transport in neurons is required for JNK pathway regulation and functional recovery in response to injury.

### Macrophages are recruited to the crush injury site and are required for functional recovery

**Fig. 7.**
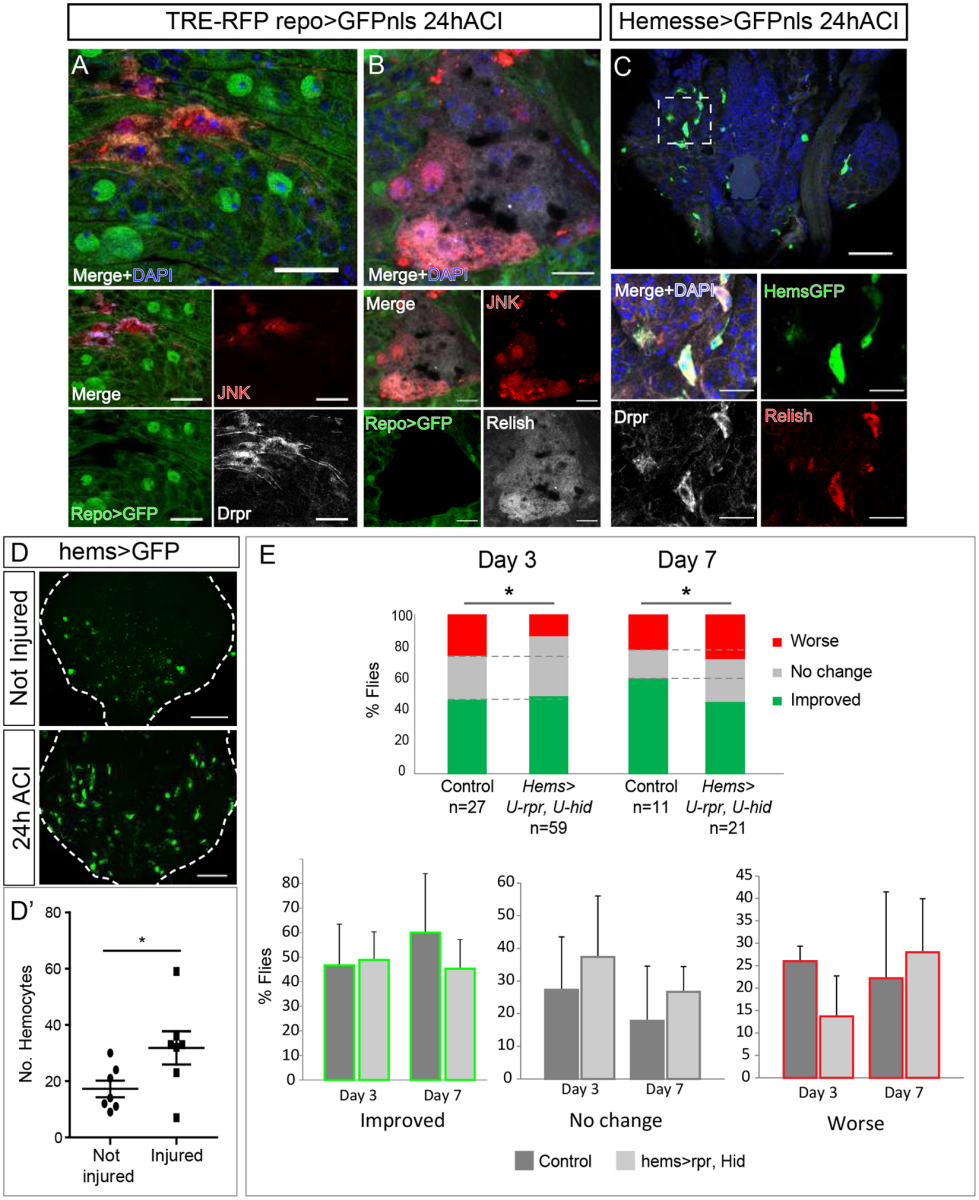
**Phagocytes are recruited to injured VNCs and required for functional recovery*.*** (A) Surface of metathoracic neuromere (MtN) stained for Drpr (grey) and active JNK signaling (TRE-RFP, red) in non glial cells. Glial cells (*repo>GFP*) are in green; nuclei were stained with DAPI (blue). The top image is a magnified version of the merged image below. (B) Surface of MtN showing non glial cells stained for Relish (grey) and active JNK signalling (red); nuclei were stained with DAPI (blue). The top image is a magnified version of the merged image below. (C) MtN hemocytes stained for Drpr (grey) and Relish (red); nuclei were stained with DAPI (blue). The boxed area is shown magnified in the four images at the bottom in A-C. (D) Representative images and quantification of hemocytes in not injured and injured MtNs. (D′) Plotted are the number of hemocytes in not injured and injured MtNs. Unpaired *t*-test, *P<0.05 (E) Quantification of the functional recovery. Percentage of flies whose hemocytes had been genetically ablated compared to that of control flies. Data were quantified for each category of movement, i.e. Worse (red), No change (grey) or Improved (green). Chi-square test with two degrees of freedom, *P<0.1. Genotypes: *TRE-RFP; repoGal4>UAS-GFPnls* (A,B). HemesseGal4>UAS-CD8GFP (C,D), CD8 is a transmembrane protein, therefore the cell surface is labeled with GFP. HemesseGal4>UAS-RedStinger (D′). *UAS-reaper, UAS-hid* (Control) and *tubGal80ts; HemesseGal4>UAS-reaper, UAS-hid* (E). Scale bars: 15 μm (A, B, C bottom image); 50 μm (C top image, D).

The immune response is crucial to facilitate the recovery process after injury in all animal systems. Previous studies have reported that the immune system plays a central role following brain injury (Gadani et al., 2015), and wound healing in epithelial tissues requires JNK signalling pathway activation (Galko and Krasnow, 2004). We report here the presence of non-glial cells [Repo negative (Repo−)] in injured VNCs, showing JNK pathway activation [positive for TRE and RFP (TRE-RFP+)] and Drpr at the injured site (Fig. 7A, Fig. S1D). These cells were located at the surface of the VNC, immediately above the outer-most glial layer. To investigate whether these cells express other genes of the immune system, we used specific antibody to analyse the amount of transcription factor Relish, the third *Drosophila* NFκB-related protein, which is homologous to mammalian NFκB1 (NFKB1) and activates the innate immune response (Tanji et al., 2010). We identified Repo−, TRE-RFP+, Relish+ cells, having a lamellocyte-like shape, above the outer-most glial cell layer (Fig. 7B). Thus, we asked whether non-glial cells that express the immune genes *drpr* and *relish* and whose JNK signalling pathway is activated are immune cells derived from the hemolymph, i.e. hemocytes, also known as macrophages in vertebrates. We used the specific hemocyte driver *Hemesse*-Gal4 to express membrane-targeted GFP tagged to CD8 (CD8-GFP) and stained injured VNCs for Drpr and Relish. We showed that GFP-positive cells (hemocytes) are recruited to the affected area within injured VNCs. These cells also stained positive for Drpr and Relish (Fig. 7C). Next, we quantified the number of hemocytes (*Hems>RedStinger*) in injured MtN compared to non-injured controls and found a significant increase within injured sites (Fig. 7D,D′). These results indicate that macrophages are recruited to the injury site in the VNC, where they activate JNK, Drpr and Relish.

Finally, we asked whether hemocytes contribute to functional recovery. We performed a functional recovery assay of injured flies and compared control flies with flies comprising genetically depleted hemocytes. To eliminate hemocytes from the organism, we overexpressed the pro-apoptotic genes *hid* and *reaper* under the control of a specific driver (Hems-Gal4>UAS-reaper, hid) – a strategy reported to eliminate all hemocytes (Lolo et al., 2012). Our results show that 3 days ACI, elimination of hemocytes does not impact recovery; on the contrary, it even improves as the percentage of worsening flies is reduced (Fig. 7E). Later, 7 days ACI, it is clear that the percentage of flies that improve locomotion is reduced when hemocytes are eliminated (Fig. 7E). So, all together the results suggest that, even though the initial response of the immune system can accelerate the worsening of flies, 7 days ACI elimination of hemocytes contributes to the worsening of flies in response to crush injury. This implies that hemocytes are required for functional recovery after crush injury.

## DISCUSSION

Glial responses to CNS injury are conserved from *Drosophila* to mammals. Here, we show that the processes that occur following CNS injury in humans (Biering-Sørensen et al., 2009; Emery et al., 1998; Kakulas, 1999; Norenberg et al., 2004) were reproduced in *Drosophila*. However, the aggressiveness of the damage in this novel paradigm is less sever compared to other models – i.e. torn-off leg, needle stuck into the brain – as this crush injury model does not imply mutilation. We present a novel paradigm that allows to study cellular events upon CNS injury in *Drosophila*, recapitulating loss of synapses, apoptosis, activation of glial immune response and the JNK pathway as observed in other injury paradigms, and providing a novel tool to study the functional regeneration of the CNS. We focused on injury and cellular responses that occur in the CNS but cannot discard that other tissues might be affected in this paradigm.

We observed that glial cells divide in response to CNS crush injury, thereby reproducing the features of *Drosophila* and other species upon injury. In the cockroach, toxin-induced glial ablation produces compensatory glial proliferation (Smith et al., 1987; Treherne et al., 1984). In vertebrates, rodents and zebrafish, spinal cord injury or toxin ablation of oligodendrocytes or oligodendrocyte progenitor cells (OPCs) induce proliferation of the remaining OPCs (Kato et al., 2018). Finally, CNS injury throughout different developmental stages (embryo and larva) also produces glial proliferation in *Drosophila* (Griffiths and Hidalgo, 2004; Kato et al., 2009, 2011; Losada-Perez et al., 2016). These studies demonstrate the high conservation of this response and validate our model to study the glial proliferative response.

**Fig. 8.**
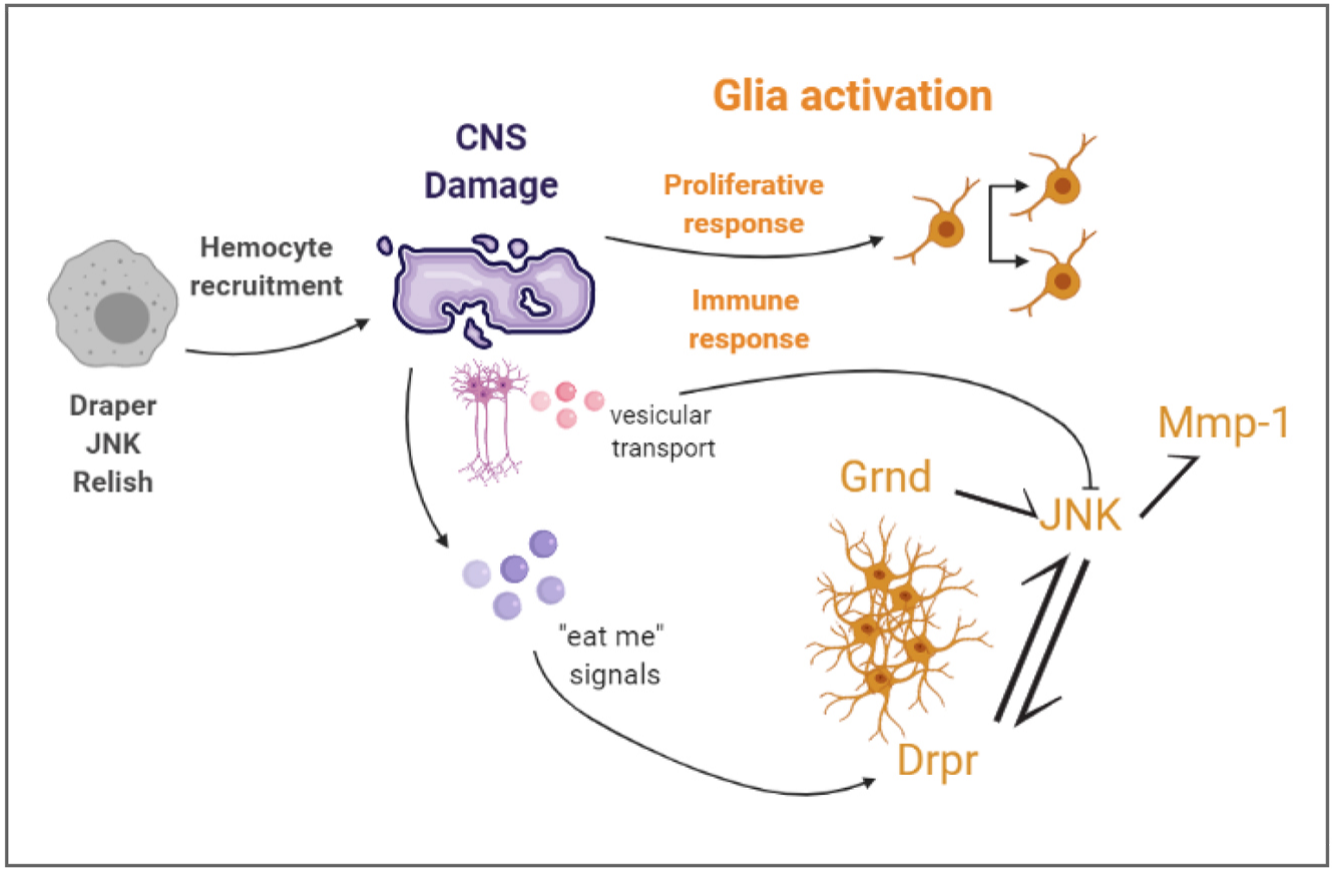
**Schematic, summarizing the cellular events described in this article**. Crush injury causes damage in the CNS, and triggers a proliferative and immune response in glial cells. The glial immune response includes activation of the JNK pathway via Grnd and Drpr. Mmp-1 activation, most probably downstream of JNK, also occurs upon crush injury. Vesicular transport in neurons is also required to modulate JNK activation upon injury. Hemocytes, containing Drpr and Relish and with activated JNK, are recruited to the injury site. Diagram created with BioRender.com.

The glial phagocytic response to injury of CNS and PNS is also conserved across species. CNS injury of adult *Drosophila* stimulates phagocytic glial cells that engulf cell debris and apoptotic cells mediated by the upregulation of the engulfment receptor *drpr/MEGF10* (Doherty et al., 2009; MacDonald et al., 2006; Morizawa et al., 2017). We showed here that *drpr* is upregulated in response to crush injury, which is in line with previous studies. Furthermore, in glial cells, Drpr activated the JNK signalling pathway that, in turn, is required to upregulate *drpr* gene expression – also in line with previous results (MacDonald et al., 2013). We demonstrated that JNK activation requires the receptor Grnd in both neurons and glia cells. Downregulation of *grnd* in neurons also reduced JNK activation following injury, highlighting the importance of neuron-glial communication. Moreover, vesicular transport in neurons is required to prevent further JNK activation in response to crush injury, supporting the significance of neuron-glial communication. These results demonstrate that, in our crush injury model, the glial immune response was activated; therefore, it can also be used to study the immune components of the GRR. We also showed the implication of circulating immune cells regarding the repair of CNS injury. Hemocytes were recruited to the injured site and are required for functional recovery. Thus, our crush injury paradigm allows the study of systemic immune responses upon CNS injury (Fig. 8).

Moreover, it enables the analysis of functional recovery and how expression of specific genes in different cell types contribute to it. Mammals and insects recover locomotion following limb amputation (Isakov et al., 2016; Kirpensteijn et al., 1999), showing that CPGs and locomotor controls are adapt to new requirements. After limb amputation flies learn to move normally (Isakov et al., 2016). Therefore, to study locomotion recovery it is required to focus on injured limbs. Our novel paradigm focussed on affected limbs and, therefore, we concentrated on the regeneration of damaged tissue. We analysed the rear legs during different time points (1, 3 and 7 days ACI), instead of following other standardised methods that measure the movement of the animal as a whole, such as during trikinetics or open-field-arena assays (Valente et al., 2007; Chiu et al., 2010; Soto-Padilla et al., 2018).

We show for the first time that wild-type flies spontaneously recover from the loss of synapses, and improve the movement of impaired legs to a certain extent. We found that, in glial cells, Drpr is required for functional recovery, linking the immune glial response to functional regeneration. *kish* knockdown in neurons reduces regenerative capacity upon injury, suggesting that vesicular transport is relevant for neuronal physiology or that neuron-glial communication is required for functional recovery itself. Previous publications (Portela et al., 2019b) suggest that RNAi of *kish* does not affect neuronal features; thus, we propose, in line with previous observations (Shklover et al., 2015), that neuron-glial communication is required for functional recovery. Finally, the fact that ablation of hemocytes – i.e. the *Drosophila* equivalent of macrophages in vertebrates – impedes functional recovery in response to crush injury, suggests that macrophages are required for functional regeneration. Together, our results demonstrate that functional recovery can be altered by genetic modifications, such as RNAi of *grnd* or *drpr* in glial cells or RNAi of *kish* neurons. It is of relevance to mention the intermediate phenotypes shown by some upstream activation sequence (UAS) *Drosophila* lines in the functional recovery results. The leakiness of the UAS system has been previously discussed (Akmammedov et al., 2017) and is associated to the residual activity of the Hsp70 minimal promoter. Therefore, even though results are consistent, some results based on the use of UAS fly lines as controls show a tendency consistent with the proposed residual activity.

A key challenge for the field is to determine the specific molecular and cellular causes of motor activity impairment and recovery; any information will facilitate the understanding of mechanisms that underlie functional regeneration. Physical damage to the nervous system causes a plethora of signals that need to be studied in-depth as a grouped response. The contribution of PI3K-GSK3 signalling pathway has been proposed to be a valid strategy to promote synaptogenesis, mitochondrial restoration and axon regeneration (Huang et al., 2019; Saijilafu et al., 2013). However, these studies mainly focused on the re-myelination process, an event crucial for the full regeneration of the nervous system but – on its own – insufficient to regenerate function upon damage. Here, we provide a new model to study physical damage of the nervous system, and determine the cellular and molecular events that are relevant for the final outcome of the organism upon injury. This paradigm of adult CNS injury brings new perspectives to this area of research, as it focuses on functional recovery, the most-relevant feature of regeneration. The advantages of the UAS/Gal4 system, the collection of specific Gal4 lines for each glial cell type, together with the ability to be able to manipulate functional recovery, make the crush-injury paradigm an ideal model in the search for new targets in order to improve CNS regeneration.

## MATERIALS AND METHODS

### Fly stocks and genetics

Experiments were performed in adult *Drosophila melanogaster* flies raised at 25°C or combining 17°C with 29°C for temporal control of *UAS/Gal-4* using the thermo-sensitive repression system *Gal80^TS^*. Stocks were generated by conventional genetics. Fly stocks used were: *UAS-CD8 GFP* (BL-79626), *UAS-myr-RFP* (BL-7119), *repo-Gal4* (BL-7415), *tub Gal80^ts^* (BL-7019), *UAS-eiger RNAi* (BL-58993), *UAS-GFP.E2f, UAS-mRFP.CycB* (Fly- FUCCI, BL-55100), *UAS-RedStinger* (BL 8546), *elavLexA* (BL-52676), *LexOP-CD8GFP* (BL-66545), *eiger MIMIC* (BL 59754), *mhc-Gal4* (BL 55133) form Bloomington *Drosophila* Stock Center; *UAS-kish RNAi* (v40884) from Vienna *Drosophila* Resource Centre; *UAS-Drpr RNAi* (BL-67034) from M. Freeman; Berlin, *UAS-puckered-LacZ, elav-Gal4* from A. Ferrus; *UAS-hid, UAS-reaper* from Carlos Estella (CBM-CSIC, Madrid, Spain); *UAS-Grnd RNAi* (v43454), *UAS-Grnd-Extra*, *TRE-RFP* (BL-59011) from Jean-Paul Vincent (The Francis Crick Institute, London, UK); *Hemese-Gal4* (BL-8699) from Dan Hultmark (Tampere University, Tampere, Sweden); *Draper Δ5* from Eduardo Moreno (Champalimaud Centre for the Unknown, Lisbon, Portugal); *eiger^1AG^*, *eiger^3AG^* from Laura Johnston (Columbia University, New York, NY).

To downregulate gene expression, we used specific UAS-RNAi lines to target the genes of interest, under the control of pan-neuronal (elav>) or pan-glial (repo>) Gal4 drivers. To control the downregulation of different genes we combined the UAS/Gal4 system with the *Tubulin-Gal80^TS^* (temperature sensitive) tool. We maintained the desired genotypes at non-permissive temperature (i.e. 17°C, at which the Gal80^TS^ repressor protein is active), to block *UAS* constructs overexpression. Then, we collected 1-2 day-old adult flies after eclosion (equivalent to 0-1 day-old flies grown at 25°C), and performed the crush injury, keeping them at permissive temperature (i.e. 29°C, at which Gal80^TS^ is inactive and the *Gal4/UAS* system is active). Injured flies dragging the third pair of legs and did not show signs of cuticle damage were selected 24 h later (24 h ACI).

### Crush injury

Flies were anesthetised on a CO_2_ plate and placed ventral side up. Each fly was immobilised using forceps. Finer forceps were used to gently apply pressure between the second and third pair of legs, aiming to injure the metathoracic VNC neuromere. Once injured, flies were placed in a fresh food vial. 24 h ACI (or 5 h ACI for experiments described in Fig. 2D); flies that dragged the third pair of legs were selected. Of those selected, flies with any sign of cuticle damage were discarded.

### BrdU incorporation

For incorporation of the thymidine analog bromodeoxyuridine (BrdU), injured flies (immediately following crush injury) and not-injured flies were placed in Eppendorf tubes containing food and BrdU (5% sugar, 1% food colorant and 1 mg/ml BrdU, Roche). Not-injured and injured flies with colorant in the abdomen were selected 24 h after crush injury.

### Immunohistochemistry and BrdU detection

Immunohistochemistry was performed following the protocol for adult *Drosophila* immunolabelling according to Purice et al. (2017). For BrdU detection, dissected VNCs were treated with 2 M HCl for 20 min (RT) prior to incubation with anti-BrdU.

We used the following antibodies: mouse anti-Repo (DSHB, 8D12 ID: AB_528448, 1:200), rat anti-BrdU (Abcam 1:500, ab6326), mouse anti-nc82 (anti-Bruchpilot; DSHB 1:30, 2ea ID: AB_2314866), rabbit anti-cleaved caspase-3 (Asp175) (5A1E) (Cell Signaling Technology, 1:50, #9664), mouse anti-Mmp1 (DSHB, 3A6B4 ID: AB_579780; 3B8D12 ID: AB_579781; 5H7B11 ID: AB_579779 used at 1:1:1, 1:50), mouse anti-Relish-C (DSHB, 21F3 ID: AB_1553772, 1:50), guinea pig anti-Repo [gift from B. Altenhein (von Hilchen et al., 2013)], rabbit anti-Drpr [gift from M. Freeman (Freeman et al., 2003)], guinea pig anti-grnd [gift from P. Leopold (Andersen et al., 2015)].

### Caspase 3 quantification

We stained adult *Drosophila* VNCs with anti-cleaved-caspase 3 (Cell Signaling Technology 1:50, #9664 lot number 18). All experimental groups were treated in parallel and analysed in blind experiments. Samples showing a bright caspase-3 signal were considered to be positive, all else as negative. The number of samples considered to be caspase-3 positive is represented as a percentage of the total number of samples.

To determine whether caspase-3 signals colocalised with condensed nuclei, we analysed the intensity of the DAPI signal by using the ‘mean grey value’ in FIJI software; this measurement takes into account both intensity and area of the signal. We measure five caspase-3-positive and five caspase-3-negative nuclei in nine samples of injured flies (i.e. 90 cells were analysed). For ROI selection of each nucleus we used ‘Oval selection’ to select the whole signal. In these confocal stacks, nuclei normally occupy two to three 1 µm slides, so we analysed the ‘mean grey intensity value’ in the slice that was closer to the centre of the nucleus, i.e. where the DAPI signal is more intense.

### Functional recovery

24 h ACI, flies were selected by the reduction of movement in the third pair of legs. We also considered the lack of external visible damage, and introduced each fly into individual food vials. Motor capacity of the third pair of legs was characterised 1, 3 and 7 days ACI. We took into account locomotion capacity, grooming and trembling phenotypes, and established distinct categories. The following categories we established from zero movement to full functionality: leg dragging without any movement (i), leg dragging with movement, flexion (ii), flexion with movement (iii), movement intention (iv), tremor or grooming intention (v), clumsy movement (vi) and complete movement (vii). For functional recovery experiments motor capacity between days 3 and 7 was compared to day 1 ACI, and registered as ‘improved’ (increased motor capacity), ‘no change’ or ‘worse’ (decreased motor capacity).

### Quantification, statistical analysis and imaging

Images were acquired by confocal microscopy (LEICA TCS SP5) and processed using Fiji (ImageJ 1.50e) software. Images were assembled using Adobe Photoshop CS4 and Adobe Illustrator CS4.

The ‘mean grey value’ was measured (using the measurement tool) by quantifying equivalent regions of interest (ROIs) of complete *z*-stack projections by using the Fiji plugin ‘sum slices’. Images were then rotated, so all VNCs had the same orientation. ROIs in different experimental groups were identical in area size and shape. To determine the ROI, we draw a rectangle around most of the hemineuromere to measure the intensity of the signal, but without any area outside the neuromere.

We calculated the ratio of metathoracic to prothoracic ganglia to normalise individual variances. In the case of anti-nc82, we found a decrease of signal in prothoracic segment in the injured samples; thus we normalised the results with the signal of anti-nc82 in the central brain.

Cells were counted using the cell counter plugin from Fiji or Imaris (Imaris 6.3.1 software- Bitplane). Quantification of Grnd protein within the membrane of glial cells and neurons was done by using the ‘Analyze→ plot profile’ tool in Fiji software.

Data were analysed and plotted using GraphPad Prism v7.0.0 or v9.0.0. Qualitative data were analysed only in Fig. 2A, where we performed a binomial test (****P<0.0001). For quantitative data, we used a D'Agostino–Pearson normality test and analysed data with normal distributions by using two-tailed *t*-test with Welch’s unequal variances *t*-test. When data had multiple comparisons, we used one-way ANOVA with Bonferroni post hoc-test; we used two-tailed Mann–Whitney U-test or Kruskal–Wallis test and Dunn’s post hoc-test for data that did not pass normality testing. The Chi-square test with two degrees of freedom was used for functional recovery experiments. Error bars represent standard deviation (+s.d.).

## Supplementary Material

Supplementary information
