# Supplementary material for: A novel injury paradigm in the central nervous system of adult Drosophila: molecular, cellular and functional aspects
Source: Dis Model Mech. 2021 Jun 1;14(5):dmm044669. doi: 10.1242/dmm.044669 (PMC8214735; doi:10.1242/dmm.044669)
Supplement: Supplementary information [file dmm-14-044669-s1.pdf]

Suppl. Figure 1

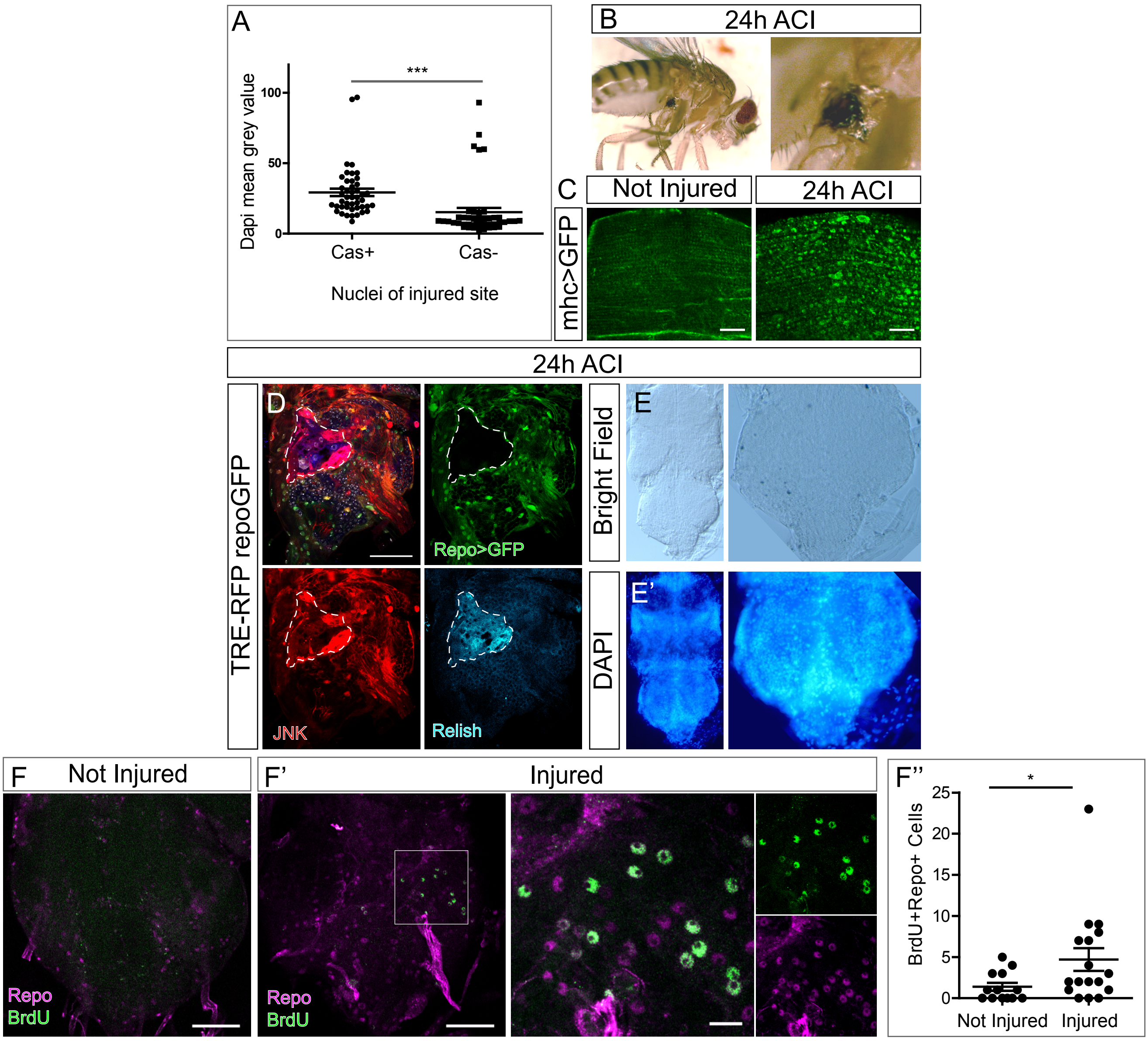

Disease Models & Mechanisms • Supplementary information

Figure S1- (A) Quantification of condensed nuclei by DAPI pixel intensity analysis of caspase-positive vs caspase-negative cells. Mann Whitney test, ***p<0.0001. **(B)** Representative image of a discarted fly with cuticle damage. **(C)** Representative image of the metathoracic dorsoventral muscle in control and injured animals. (**D**) Metathoracic injured VNC with disrupted BBB. (**E**) Image of the macroestructure of an injured VNC in bright field microscopy image and DAPI. **(F)** Representative Metathoracic neuromeres showing the BrdU pulse experiments. Quantification of repo+ (magenta) BrdU+ (green) cells. Unpaired T-test, *p<0.05**.** Genotypes: **A, B, E** and **F**, *wt*; **C**, *mhcGal4>UAS-CD8-GFP*; **D**, *TRE-RFP; repoGal4>UAS-GFPnls.* Scale bar: A, B and F, 50 µm. D , 15 µm. F', 50 µm and 15 µm.

Disease Models & Mechanisms • Supplementary information

Suppl. Figure 2

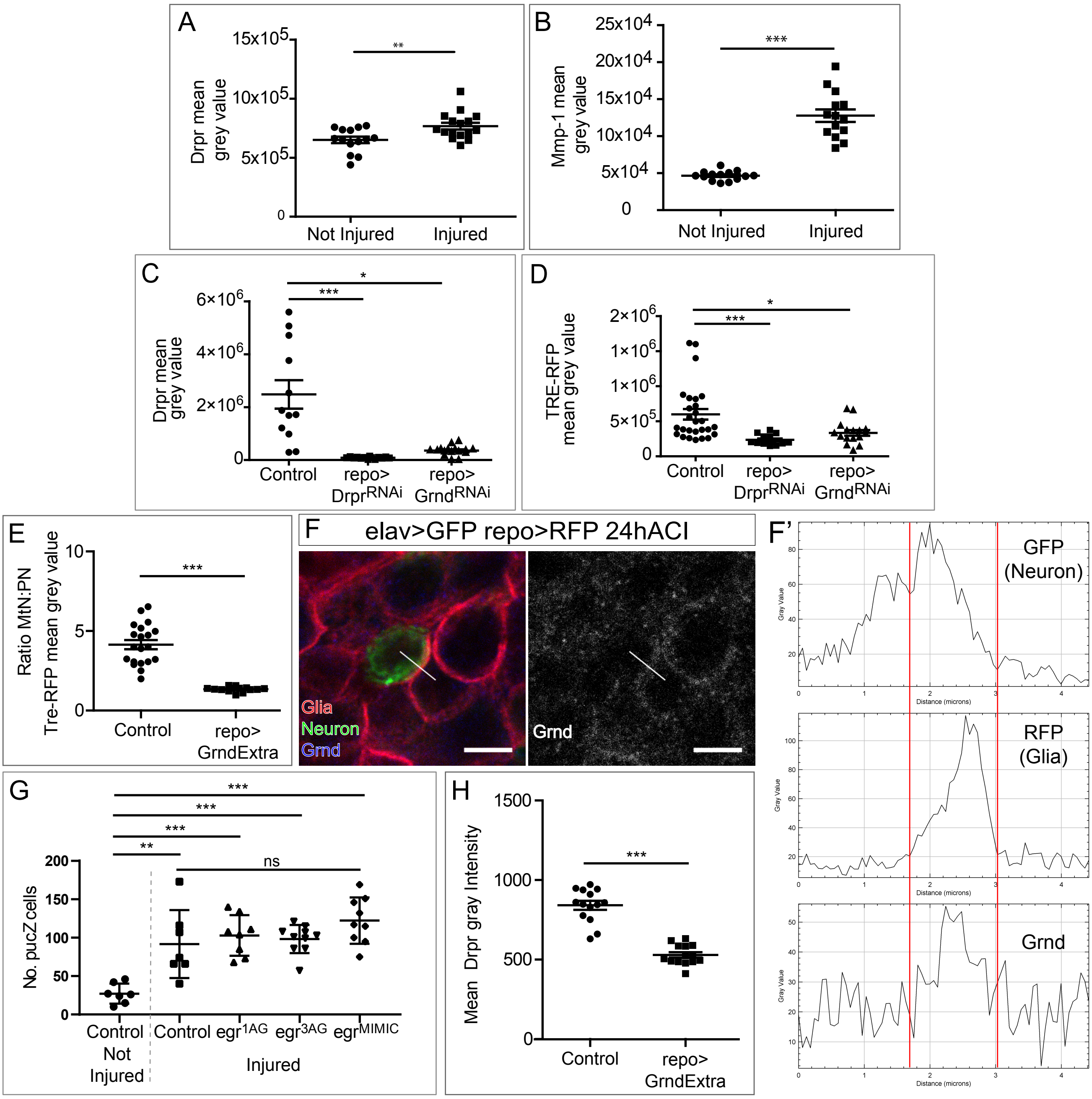

Disease Models & Mechanisms • Supplementary information

Figure S2- (A) Quantification of Drpr staining in methatoracic neuromeres of not injured and injured (5hACI) VNCs. Unpaired T-test, **p<0.001. **(B)** Quantification of Mmp-1 staining in methatoracic neuromeres of not injured and injured (24hACI) VNCs. Unpaired T-test, **p<0.001. **(C-D)** Quantification of Drpr staining and TRE-RFP signal in methatoracic neuromeres of injured controls and knocking down *drpr* or *grnd*. Kruskal- Wallis test, P<0.0001. Dunn's Multiple Comparison Test, *** P<0.001, *p<0.05. **(E)** Quantification of Ratio MtN/PN of TRE-RFP signal of injured controls and knocking down *grnd* with Grnd-EXTRA. Unpaired T-test, ***p<0.0001. **(F)** Representative image of Grnd staining (blue) in neuron (green) and glial cells (red) and Grnd alone (grey). **(F')** Plot profile of Grnd signal and glia and neuron membrane. **(G)** Number of puc-Z positive cells in not injured controls, injured controls and injured *eiger* mutants. Ove-Way ANOVA, p<0.0001 and Bonferroni multiple comparison, **p<0.005, *** p<0.0001. **(H)** Quantification of Drpr staining of injured controls and knocking down *grnd* with Grnd-Extra. Unpaired T-test, ***p<0.0001. Genotipes: **A** and **B**, wt. **C** and **D**, *TRE-RFP; repoGal4-UAS-GFPnls*, *TRE-RFP; repoGal4>UAS-GFPnls, UAS-drprRNAi*, *TRE-RFP; repoGal4>UAS-GFPnls, UAS-grndRNAi*. **E** and **H**, TRE-*RFP; +, UAS-grndEXTRA* (Control) and *TRE-RFP; repoGal4>UAS-GFPnls, UAS-grndEXTRA*. ***F,*** *elavLexA>lexAOP-GFP; repoGal4>UAS-myr-RFP.* **G**, *puckered-LacZ/+, eiger1AG/ eiger1AG; puckered-LacZ/+, eiger3AG/ eiger3AG; puckered-LacZ/+* **,** *eigerMIMIC/ eigerMIMIC; puckered-LacZ/+.* Scale bar: F, 5 µm.

Disease Models & Mechanisms • Supplementary information

Supplementary Figure 3

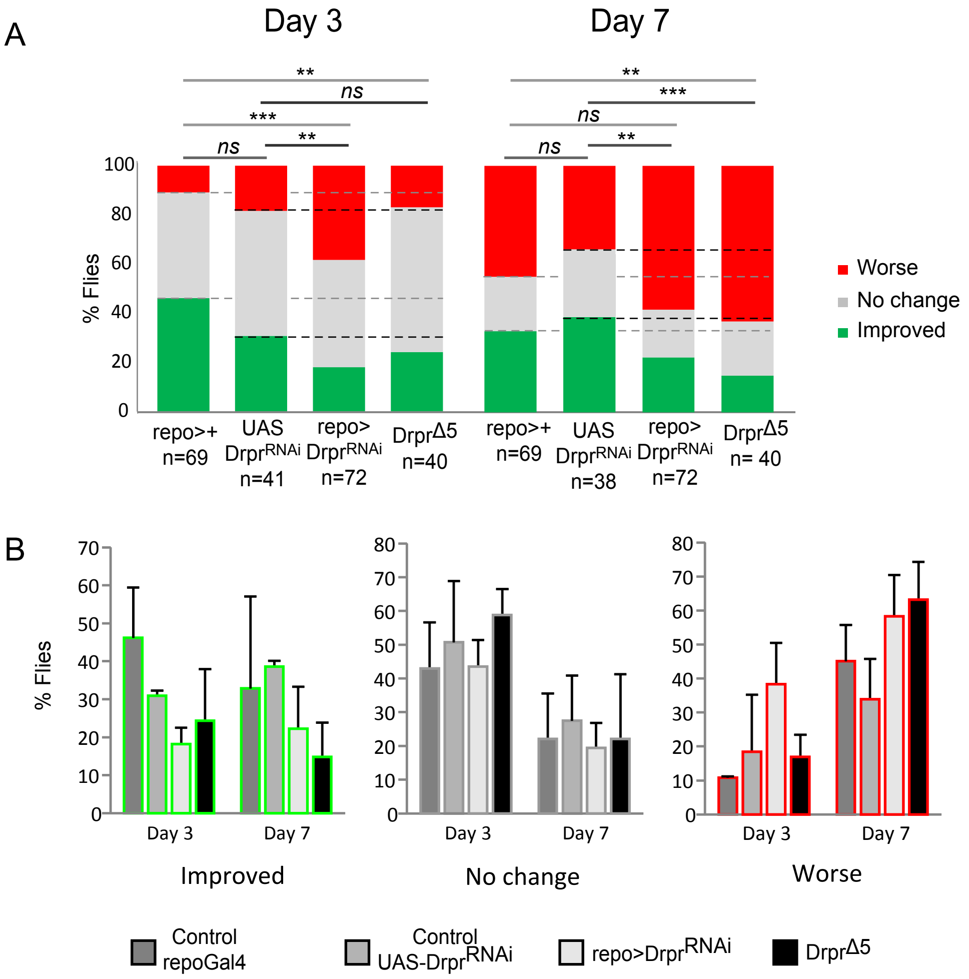

Figure S3- (A) Histograms represent the percentage of injured flies corresponding to each of the three categories of movement, Worsen (red), No change (grey) or Improve (green), compared to T0 (24h ACI). Functional recovery capacity of control flies (*repoGal4* and *UAS-drprRNAi* alone) compared with *drpr* knocked down in glia and *drpr* mutants. Chi-square test with 2 degrees of freedom, ***p<0.0001, **p<0.005**.** **(B)** Histograms represent the percentage of flies corresponding to each category separately. Genotypes: *tubGal80ts; repoGal4/+*, UAS *DrprRNAi/+, tubGal80ts, repoGal4>UAS- DrprRNAi,* and *DrprΔ5/ DrprΔ5.*

Disease Models & Mechanisms • Supplementary information

Supplementary Figure 4

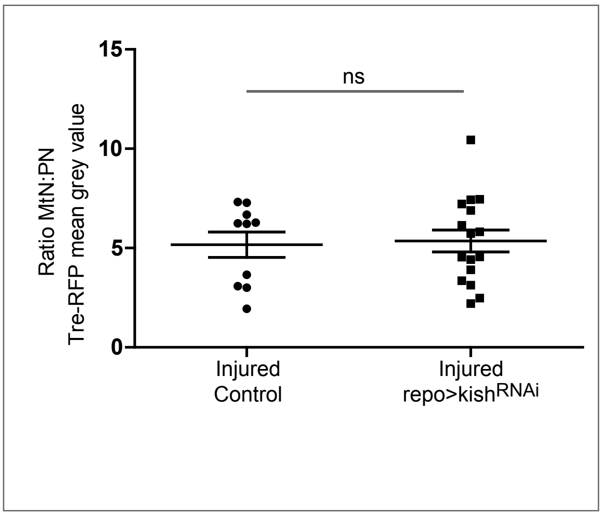

Figure S4 - Vesicular transport in glia does not affect JNK activation upon injury. Quantification of TRE-REF mean grey value ratio MtN/NP in injured control flies and injured flies where vesicular transport in glia is blocked. Unpaired T-test, n.s. Genotypes: *TRE-RFP/TubGal80ts; +, UAS-kishRNAi* (Control) and *TRE-RFP; repoGal4>UAS-GFPnls, UAS-kishRNAi.*

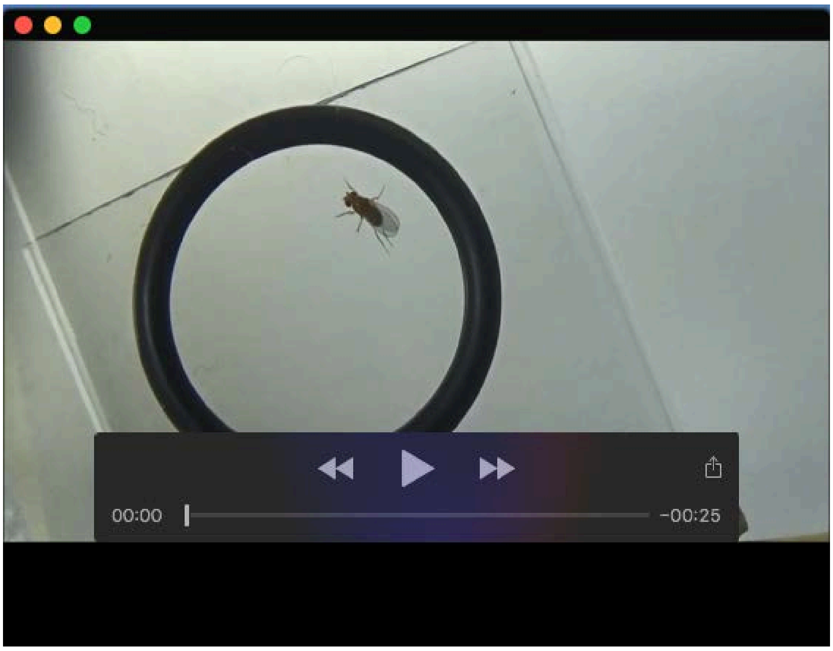

Movie 1. Representative movie of an injured fly 24h ACI. Flies that dragg the posterior pair of legs are selected as damaged. 72h ACI there is a spontaneous recovery of motility in this posterior pair of legs

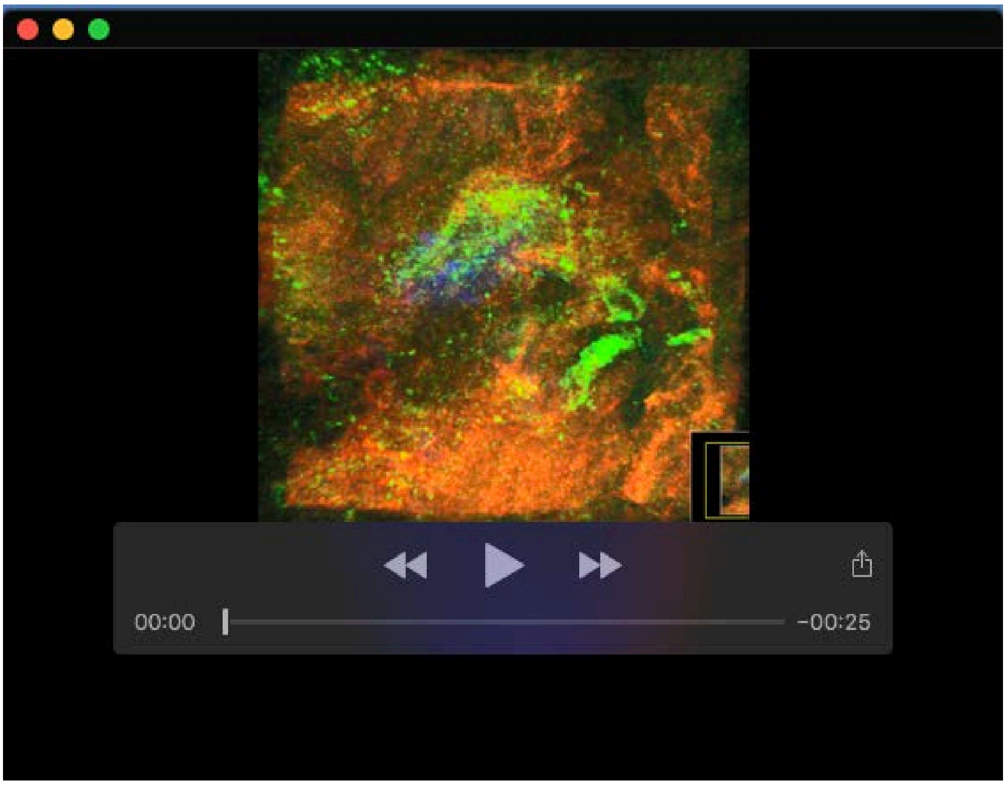

Movie 2. 3D reconstruction of confocal images of adult CNS 24 ACI. Drpr signal (green) co-ocalizes with glial membrane (red). These glial membranes are surrounding caspase-3-positive bodies (blue). Digitalization was done using Imaris (bitplane).
